# Azoxystrobin Induces Apoptosis of Human Esophageal Squamous Cell Carcinoma KYSE-150 Cells through Triggering of the Mitochondrial Pathway

**DOI:** 10.3389/fphar.2017.00277

**Published:** 2017-05-17

**Authors:** Xiao-ke Shi, Xiao-bo Bian, Tao Huang, Bo Wen, Ling Zhao, Huai-xue Mu, Sarwat Fatima, Bao-min Fan, Zhao-xiang Bian, Lin-fang Huang, Cheng-yuan Lin

**Affiliations:** ^1^Yunnan Minzu University-Hong Kong Baptist University, Joint Laboratory of Traditional Natural Medicine, Yunnan Minzu UniversityKunming, China; ^2^Lab of Brain and Gut Research, School of Chinese Medicine, Hong Kong Baptist University, Kowloon TongHong Kong SAR, China; ^3^Graduate School, New York University, New YorkNY, United States; ^4^Institute of Medicinal Plant Development, Peking Union Medical College and Chinese Academy of Medical SciencesBeijing, China

**Keywords:** azoxystrobin, human esophageal squamous cell carcinoma, apoptosis, mitochondrial pathway, anti-tumor

## Abstract

Recent studies indicate that mitochondrial pathways of apoptosis are potential chemotherapeutic target for the treatment of esophageal cancer. Azoxystrobin (AZOX), a methoxyacrylate derived from the naturally occurring strobilurins, is a known fungicide acting as a ubiquinol oxidation (Qo) inhibitor of mitochondrial respiratory complex III. In this study, the effects of AZOX on human esophageal squamous cell carcinoma KYSE-150 cells were examined and the underlying mechanisms were investigated. AZOX exhibited inhibitory effects on the proliferation of KYSE-150 cells with inhibitory concentration 50% (IC_50_) of 2.42 μg/ml by 48 h treatment. Flow cytometry assessment revealed that the inhibitory effect of AZOX on KYSE-150 cell proliferation occurred with cell cycle arrest at S phase and increased cell apoptosis in time-dependent and dose-dependent manners. Cleaved poly ADP ribose polymerase (PARP), caspase-3 and caspase-9 were increased significantly by AZOX. It is worth noted that the Bcl-2/Bax ratios were decreased because of the down-regulated Bcl-2 and up-regulated Bax expression level. Meanwhile, the cytochrome *c* release was increased by AZOX in KYSE-150 cells. AZOX-induced cytochrome *c* expression and caspase-3 activation was significantly blocked by Bax Channel Blocker. Intragastric administration of AZOX effectively decreased the tumor size generated by subcutaneous inoculation of KYSE-150 cells in nude mice. Consistently, decreased Bcl-2 expression, increased cytochrome *c* and PARP level, and activated caspase-3 and caspase-9 were observed in the tumor samples. These results indicate that AZOX can effectively induce esophageal cancer cell apoptosis through the mitochondrial pathways of apoptosis, suggesting AZOX or its derivatives may be developed as potential chemotherapeutic agents for the treatment of esophageal cancer.

## Introduction

Azoxystrobin (AZOX) is a methoxyacrylates derived from the naturally occurring strobilurins (Abdelraheem et al., 2015) and commonly used as a systemic fungicide in agriculture. It exerts its fungicidal activity by inhibiting the ubiquinol oxidation (Qo) center of fungal respiration complex III through cytochrome pathway (Bartlett et al., 2002). Chronic AZOX treatment was reported to reduce the body weight of the mice and inhibit *de novo* lipogenesis in HepG2 cells through AMP-activated protein kinase (AMPK) signaling (Gao et al., 2014).

Mitochondrial respiration is a complex of metabolic reactions to provide the universal energy adenosine triphosphate (ATP) in the cells via oxygen consumption process, which has recently emerged as one of the strategies in cancer therapies (Jose and Rossignol, 2013; Viale et al., 2015). Based on current understanding and evidence, mitochondrial functions is essential for tumor initiation, growth, invasion and metastasis (Enns and Ladiges, 2012; Amoedo et al., 2014; Tan et al., 2014). The mitochondrial complex-III inhibitor, mahanine, could effectively suppress cell proliferation by inducing G0/G1 phase arrest in human glioblastoma multiforme cells (Bhattacharya et al., 2014). Recently, an Food and Drug Administration (FDA) approved anthelmintic drug, pyrvinium was demonstrated to induce lymphoma T-cell apoptosis in mitochondrial respiration-dependent manner (Xiao et al., 2016). Moreover, inhibition of mitochondrial respiration by As_2_O_3_ showed a great potential to enhance drug-induced apoptosis in human leukemia cells (Pelicano et al., 2003). These findings raise the possibility that mitochondrial respiration pathway can be a therapeutic target to explore drugs for cancer treatment.

Recent studies revealed that enhanced mitochondrial respiration may be involved in the radioresistance of esophageal adenocarcinoma (EAC) by *in vitro* studies with consistent observations in EAC patients (Lynam-Lennon et al., 2014). Considering the close correlations between mitochondrial respiration and tumor cell growth, we hypothesize that AZOX is a potential candidate for esophageal cancer drug exploration.

In this study, we determined the *in vitro* and *in vivo* anticancer effects of AZOX on representative esophageal squamous carcinoma cell line KYSE-150 (Shimada et al., 1992) with an emphasized investigation on the mitochondrial apoptosis pathway.

## Materials and Methods

### Cell Culture

The esophageal cancer cell lines KYSE-150, KYSE-70, and KYSE-450 were gifted by Dr. Johnny C.O. Tang of Hong Kong Polytechnic University (Hong Kong). KYSE-150 cell were cultured in Roswell Park Memorial Institute (RPMI) 1640 supplemented with 2% fetal bovine serum (FBS) while KYSE-70 and KYSE-450 cell lines were grown in RPMI 1640 supplemented with 10% FBS (Shimada et al., 1992). HCT116, SW480, Huh-7, HepG2, and MIHA cell lines were purchased from American Type Culture Collection (ATCC, Manassas, United States). Cells were cultured in Dulbecco’s Modified Eagle’s Medium (DMEM) supplemented with 10% FBS according with the instruction. All the culture medium was supplemented with 100 units/ml penicillin and 100 μg/ml streptomycin (Thermo Fisher Scientific Inc., MA, United States). Cells were maintained in a humidified atmosphere containing 5% CO_2_ at 37°C.

### Chemicals and Reagents

**FIGURE 1:**
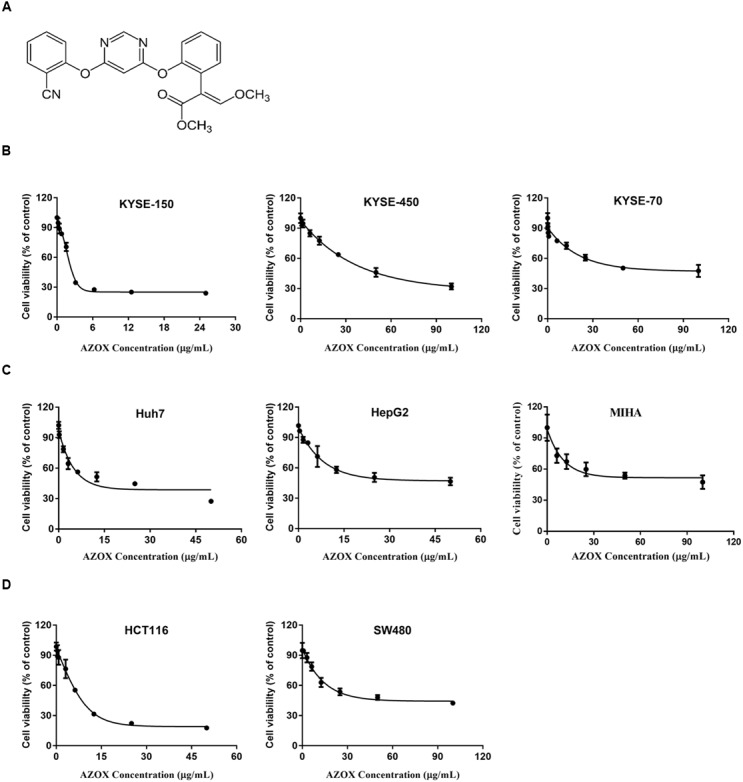
**AZOX decreased the viability of cancer cells. (A)** Chemical structure of AZOX. **(B)** MTT assay of esophageal cancer cell lines (KYSE-150, KYSE-450, and KYSE-70) treated with increasing concentration of AZOX for 48 h. **(C)** MTT assay of liver cancer cell lines (Huh-7 and HepG2) and non-tumorigenic normal human hepatocyte cell line (MIHA) treated with increasing concentration of AZOX for 48 h. **(D)** MTT assay of colon cancer cell lines (HCT116 and SW480) treated with increasing concentration of AZOX for 48 h. The IC_50_ values were calculated using GraphPad Prism 6.0 software.

AZOX (illustrated in **Figure 1A**) was purchased from Sigma–Aldrich (MO, United States) and dissolved in dimethyl sulfoxide (DMSO) (Sigma–Aldrich, MO, United States) with stock concentration at 25 mg/ml and stored in -20°C. Bax channel blocker was purchased from Tocris Bioscience (Bristol, United Kingdom). Antibodies for β-actin, cleaved caspase-3, cleaved caspase-9, cleaved caspase-8, β-actin, Bcl-2, Bax, Bad, and cleaved-poly ADP ribose polymerase (PARP) were purchased from Cell Signal Technology (MA, United States). The reagents for mitochondrial protein extraction were purchased from Merck Millipore (MA, United States).

### MTT Assay

The effects of AZOX on cell proliferation and viability of different cell lines were assessed by 3-(4,5-dimethylthiazol-2-yl)-2,5-diphenyltetrazolium bromide (MTT) assay. MTT powder was purchased from Sigma–Aldrich (MO, United States). Briefly, cells with a density of 3000 cells/well was seeded in 96-well plates 24 h prior to AZOX treatment. Then the medium was removed and cells were exposed to different concentration of AZOX for another 48 h followed by MTT assay. Independent experiments were performed in triplicate.

### Cell Cycle Analysis

The cell cycle phase distribution was determined by fluorescence-activated cell sorting (FACS) analysis of cellular DNA content. KYSE-150 cells were treated with AZOX in different dosages and time points. Then cells were dissociated with 0.05% trypsin-ethylenediaminetetraacetic acid (EDTA) (Thermo Fisher Scientific Inc., MA, United States), washed with cold phosphate-buffered saline (PBS), and fixed in 70% ethanol at 4°C overnight. The fixed cells were washed twice with PBS and stained with propidium iodide (PI) working buffer (PI 50 μg/ml, RNase 2.5 μg/ml) in the dark at room temperature for 1 h. The stained cells were assessed by flow cytometry (Beckman Coulter, CA, United States) and analyzed with ModFit 4.0 software.

### Cell Apoptosis Analysis

The pro-apoptosis effect of AZOX on KYSE-150 was accessed with FITC Annexin V Apoptosis Detection Kit (BD Biosciences, San Jose, CA, United States) following the standard protocol. Briefly, cells were treated with AZOX with different concentration or time points, and dissociated into single cells with 0.05% trypsin. After washing twice with PBS, cells were stained with annexin V/PI for 20 min at room temperature in the dark and then detected by flow cytometry (Beckman Coulter, United States). The percentage of apoptosis cells were expressed as: (a) early apoptosis cancer cells (annexin +ve; PI -ve), (b) late apoptosis cancer cells (annexin +ve; PI +ve) (Paul et al., 2013).

### Western Blot Analysis

KYSE-150 cells treated with AZOX for different concentration or different time course. Whole cell lysates were obtained by suspending the cells with pre-cold RIPA lysis buffer (50 mM Tris–HCl, 0.1% SDS, 150 mM NaCl, 2 mM EDTA, 50 mM NaF, 0.5% sodium deoxycholate, 1% NP-40, pH = 7.4) for 30 min on ice, followed by centrifugation at 14,000 rpm for 10 min at 4°C. To detect the expression level of Bax, Bcl-2, and Bad, mitochondria fractions were extracted. Briefly, cells were collected and washed with ice-cold PBS, centrifuged at 600 ×*g* for 5 min. The cell pellets were resuspended and homogenized in 1× cytosol extraction buffer mix. After centrifugation steps, the supernatant was isolated as cytosolic fraction. The cell pellets were then dissolved in mitochondria extraction buffer mix and vortex for 10 s to obtain the mitochondrial fraction. Western blot analysis was conducted as described previously (Lin et al., 2015). Protein concentration was detected using Pierce BCA protein assay kit (Thermo Fisher Scientific Inc, MA, United States), subjected to 10% sodium dodecyl sulfate-polyacrylamide gel electrophoresis (SDS-PAGE) and transferred to polyvinylidene difluoride membranes. After blocking with 5% bovine serum albumin in PBS-Tween 20 buffer (PBST) for 1 h at room temperature, the membranes were incubated with the primary antibodies overnight at 4°C. After three-time washing with PBST, the membranes were re-incubated with corresponding secondary antibodies for 1 h at room temperature and then subjected to electrochemiluminescence immunoassay.

### Xenograft Studies

Male BALB/c nude mice (6-week-old) were purchased from the Laboratory Animal Services Centre, The Chinese University of Hong Kong. The mice were bred in barrier facilities with 12 h light/dark cycle environment and free access to food and water *ad libitum*. Each mouse was inoculated subcutaneously on the flank of the mice with 2 × 10^6^ KYSE-150 cells in 100 μl PBS. Once tumor size reached about 80 mm^3^, the mice were divided randomly into two groups with six mice per group: AZOX group received AZOX (40 mg/kg/day) dissolved in 0.5% carboxymethylcellulose sodium solution by intragastric administration, while vehicle group received daily 0.5% carboxymethylcellulose sodium solution by intragastric administration. Tumor volume was measured with calipers and calculated by width^2^× length × 0.4 as previously described (Chen et al., 2015). General health and body weight were also monitored every 2 days. The mice were anesthetized with 7% chloral hydrate and sacrificed 14 days later, and the tumor xenografts were dissected, weighed and fixed in 4% Paraformaldehyde (PFA) for further examination. All experimental protocols were approved by The Animal Ethics Committees of Hong Kong Baptist University, and in accordance with “Institutional Guidelines and Animal Ordinance” from Department of Health, Hong Kong Special Administrative Region.

### Statistical Analysis

The data was evaluated as means ± standard error of the mean (SEM). Statistical differences between individual groups were evaluated using Student’s *t*-test or one-way analysis of variance (ANOVA). All experiments were performed at least three times independently. GraphPad Prism 6.0 software (GraphPad Software Inc., San Diego, CA, United States) was used for the calculations, and *p* < 0.05 was considered statistically significant.

## Results

### AZOX Inhibited KYSE-150 Cell Proliferation

The effect of increasing AZOX concentrations in cancer cells was examined by MTT assay. The results showed that the cytotoxicity of AZOX on cancer cells are cell-type dependent after 48 h treatment. As shown in **Figure 1B**, in esophageal cancer cell lines, the inhibitory concentration 50% (IC_50_) values were calculated to be 2.42, 40.76, and 44.88 μg/ml for KYSE-150, KYSE-450, and KYSE-70, respectively. In contrast, KYSE-520 cell line did not show any effect after AZOX treatment (data not shown). In hepatocellular carcinoma cancer cell lines, AZOX did not alter the Hep3B cell proliferation but could decrease HuH-7 and HepG2 cell viability with IC_50_ values of 10.86 μg/ml and 22.52 μg/ml, respectively (**Figure 1C**). It is worth noted that AZOX inhibit MIHA cell growth with IC_50_ of 68.87 μg/ml. In parallel experiments on colon cancer cell lines, HCT116 cell were more sensitive to AZOX with IC_50_ at 8 μg/ml than SW480 cell (IC_50_, 45.44 μg/ml) (**Figure 1D**).

### AZOX Induced KYSE-150 Cell Cycle Arrest in the S Phase

**FIGURE 2:**
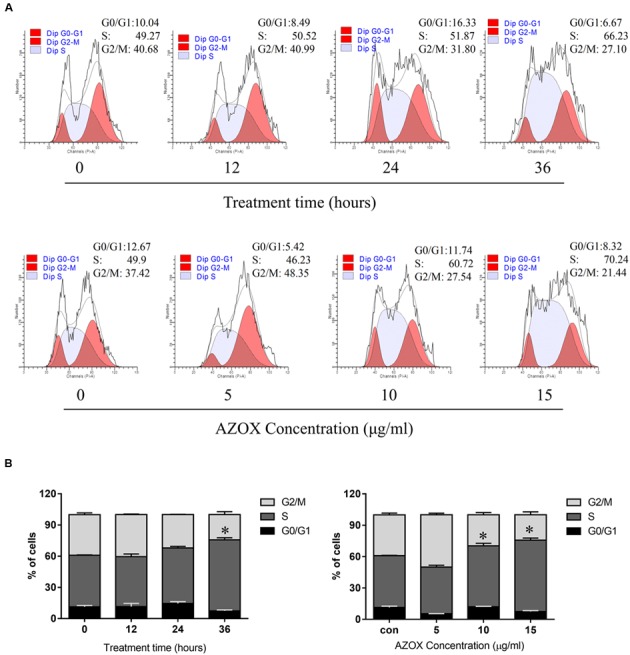
**AZOX induced KYSE-150 cell cycle arrest. (A)** Cells were treated with AZOX (15 μg/ml) time-dependently and then with indicated doses of AZOX for 36 h. Flow cytometry cell cycle analysis of KYSE-150 cell cycle arrest with AZOX treatment for different concentration or different time point were then performed. **(B)** Statistical charts of G0/G1, S and G2/M phase. All data are represented as means ± SEM of three independent experiments (^∗^*p* < 0.05).

To test whether AZOX can disturb cell cycle distribution, the effect of AZOX was tested in KYSE-150 cells by PI staining, followed by FACS analysis. Cell treated with DMSO were used as control. As shown in **Figures 2A,B**, the cells treated with AZOX (15 μg/ml) resulted in an apparent increase in the proportion of S phase at 36 h after the exposure. Further, KYSE-150 cells were treated with AZOX at concentration of 5, 10, and 15 μg/ml. FACS analysis were performed 36 h after the treatment. Significant difference of S phase cell proportion was observed at the dose of 10 μg/ml, and more effective at 15 μg/ml.

### AZOX Induced KYSE-150 Cell Apoptosis

**FIGURE 3:**
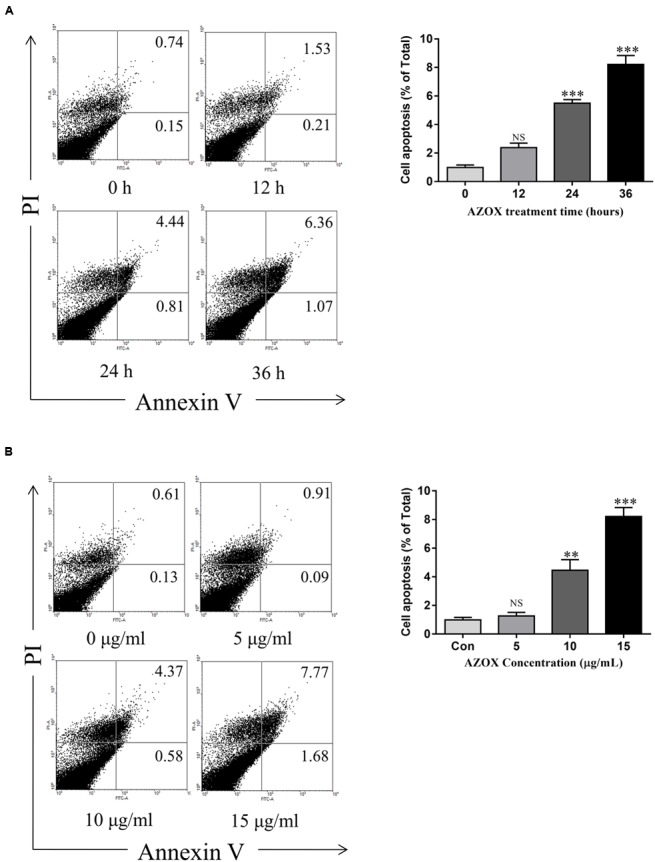
**AZOX induced KYSE-150 cell apoptosis. (A)** Cells were treated with AZOX (15 μg/ml) time-dependently. **(B)** Cells were treated with AZOX dose-dependently. Flow cytometry cell apoptosis analysis were then performed. Statistical charts of the apoptosis cells were illustrated. All data are represented as means ± SEM of three independent experiments (NS: not significant, ^∗^*p* < 0.05, ^∗∗^*p* < 0.01, and ^∗∗∗^*p* < 0.001).

Apoptosis is a known cell process linked with mitochondrial respiration (Pelicano et al., 2003). To test the pro-apoptosis effect of AZOX, KYSE-150 cells were treated with AZOX at different time points or with different concentrations. After staining with annexin V/PI, the cells were subjected to quantitative analysis of the apoptotic cell percentage. The result indicated that AZOX could induce cell apoptosis time dependently and dose dependently (**Figures 3A,B**). The shorted effective time was 24 h and the minimal effective concentration was 10 μg/ml. Treatment of 15 μg/ml AZOX for 36 h can induce approximate 10% cell apoptosis.

### AZOX Induced Cell Apoptosis of KYSE-150 via Mitochondrial Pathway

**FIGURE 4:**
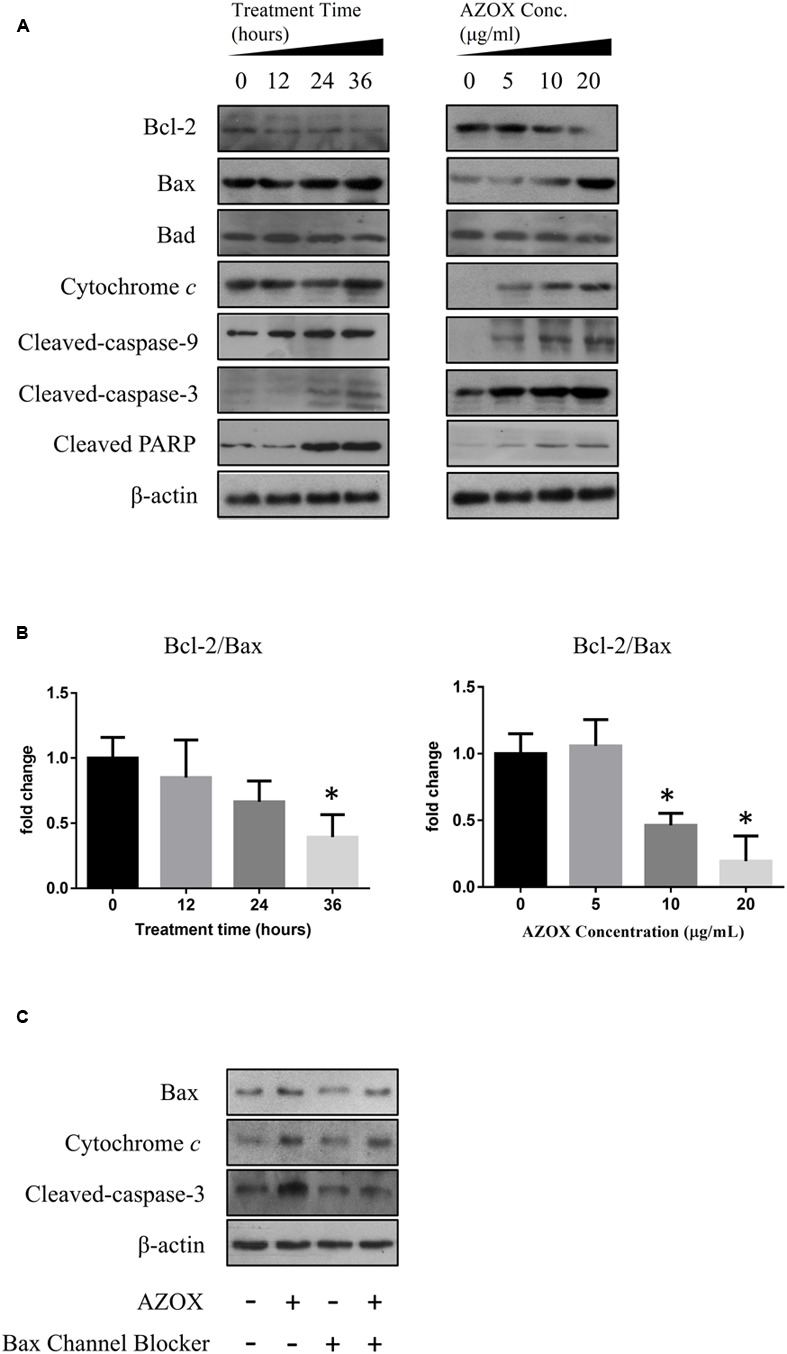
**AZOX induced KYSE-150 cell apoptosis through by activation of the intrinsic pathway.** KYSE-150 cells were treated with 20 μg/ml AZOX for 12, 24, and 36 h, respectively, or cultured in the presence of 10, 15, and 20 μg/ml AZOX for 36 h. Cells were then washed, collected with lysis buffer, and subjected to SDS-PAGE. **(A)** The expression of cleaved PARP, Bcl-2, Bax, Bad, cytochrome *c*, and the cleaved caspases were detected by western blot analysis. **(B)** Time-dependent and dose-dependent effects of AZOX on the ratio of Bcl-2/Bax. **(C)** KYSE-150 cells were pretreated with Bax channel blocker for 30 min, and then subjected to 36 h treatment of AZOX (20 μg/ml). The samples were collected and detected by western blot analysis. Data are represented as means ± SEM of three independent experiments (^∗^*p* < 0.05).

To investigate the underlying mechanisms of AZOX-induced apoptosis of KYSE-150 cell, the apoptotic proteins were measured by western blot analysis after the treatment with AZOX at different time points or with different concentrations. Cleavage of PARP in KYSE-150 cells, the indicator of cell apoptosis, was increased by AZOX both time-dependently and dose-dependently (**Figure 4A**). Since PARP is the critical substrate of caspase family proteins, we next detected whether AZOX would further affect the activities of caspase-3 and caspase-9. As shown in **Figure 4A**, the cleavage of caspase-3 and caspase-9, showed time-dependent and dose-dependent increases, indicating that AZOX induced KYSE-150 cell apoptosis via activating the caspase-3 and caspase-9. Interestingly, the expression of cleaved caspase-8 was significantly increased by AZOX in KYSE-150 cells (Supplementary Figure 1A). Furthermore, we explored the effect of AZOX on the apoptosis-related proteins in intrinsic mitochondrial pathway. The pro-survival protein Bcl-2 showed slightly decrease by AZOX with a corresponding increased level of Bax (**Figure 4A**). As a result, the ratio of Bcl-2 to Bax (Bcl-2/Bax) was significantly decreased in KYSE-150 cells after 36 h exposure to AZOX with the concentration of 10 and 20 μg/ml (**Figure 4B**). Non-phosphorylated Bad, however, was not significantly affected by AZOX *in vitro* (**Figure 4A**). Cytochrome *c* released from the mitochondria has been proposed to be a potential event which would trigger caspase-3, 7/9-dependent cell apoptosis (Jiang and Wang, 2000; Li et al., 2000). In our results, the expression of cytochrome *c*, unsurprisingly, was increased after AZOX treatment in a time-dependent and dose-dependent manner (**Figure 4A**). In parallel studies, Bax channel blocker could effectively block AZOX-induced cytochrome *c* expression and caspase-3 activation (**Figure 4C**).

### AZOX Inhibited the *In Vivo* Tumor Growth

**FIGURE 5:**
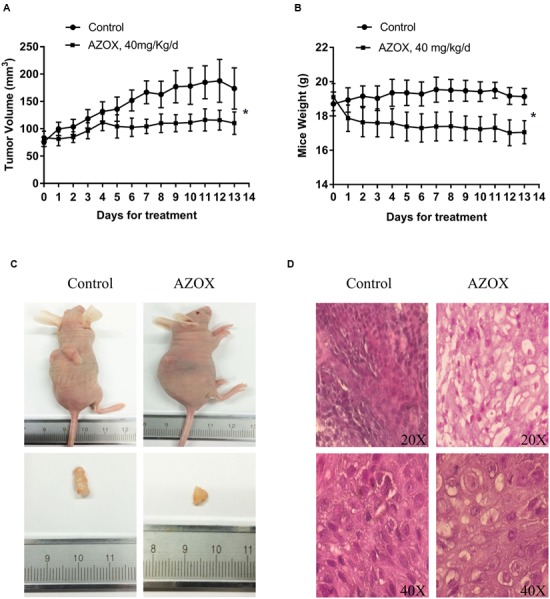
**AZOX inhibited the *in vivo* tumor growth in nude mice.** Nude mice transplanted with KYSE-150 xenografted tumors were intragastric administrated with vehicle or AZOX 40 mg/kg/day for 13 days. **(A)** The growth curves of tumor volume between the control group (*n* = 6) and AZOX group (*n* = 6). **(B)** Comparison of the mice body weight between control and AZOX group. The weight of the nude mice was weighted and recorded every day from the 1st day of AZOX treatment. **(C)** The representatives of the control and AZOX-treated mice and tumors. At the end of the experiment, mice were anesthetized with chloral hydrate, the tumor and the mice were weighted and photographed. **(D)** Representative hematoxylin and eosin (H&E) staining of the xenografted tumors of control and AZOX group. The tumors were dehydrated, fixed, and sectioned, and then stained with H&E to identify the proliferation of the cancer cells.

**FIGURE 6:**
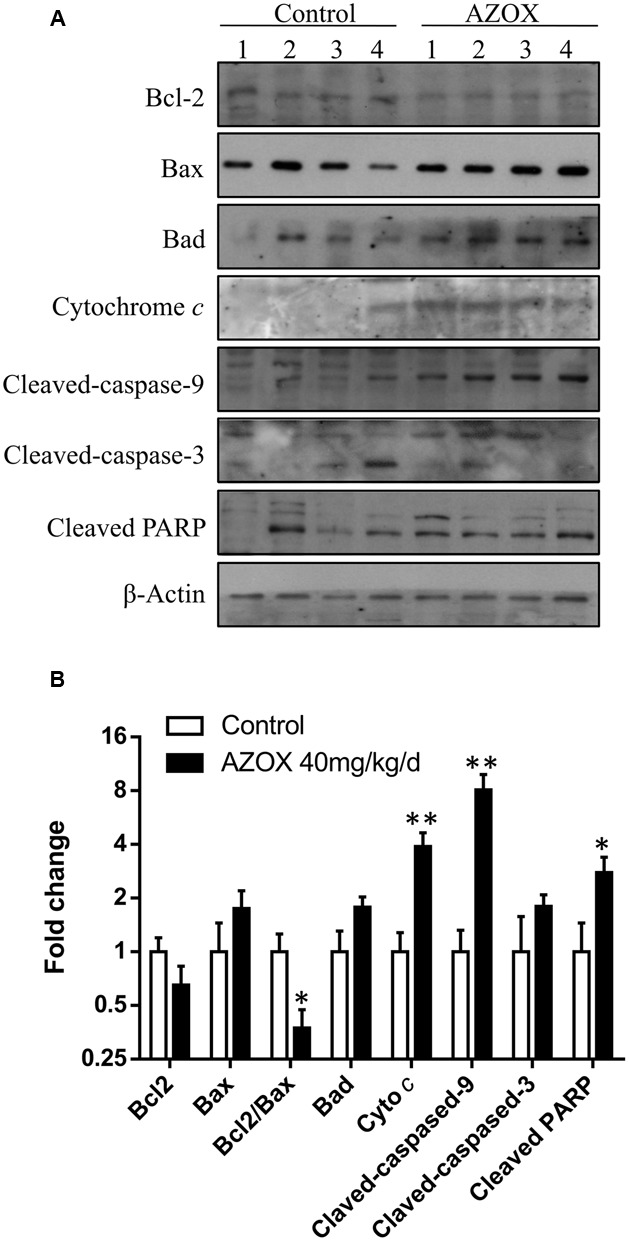
**AZOX abrogates the *in vivo* tumor growth through activating the intrinsic apoptosis pathway. (A)** The tumors dissected from the nude mice were homogenated and subjected to the total protein extraction followed by western blot analysis to detect the expression of cleaved PARP, Bcl-2, Bax, Bad, cytochrome *c*, and the cleaved caspases. **(B)** Statistical charts of the western blot results. All data are represented as means ± SEM of three independent experiments (^∗^*p* < 0.05 and ^∗∗^*p* < 0.01).

The *in vivo* anti-cancer effect of AZOX was assessed in tumor xenografted mouse model. KYSE-150 cells were injected on the flank of the mice subcutaneously to reach about the size of 80 mm^3^, then the mice received 40 mg/kg/day AZOX or carboxymethylcellulose sodium solution for 14 consecutive days. The mice body weight and tumor volume were recorded every day. AZOX treatment significantly retarded tumor growth in nude mice as shown in **Figure 5A** (*p* = 0.003) and **Figure 5C**. Accordingly, compared to the control group, mice body weight in AZOX group was significantly decreased (*p* = 0.0125; **Figure 5B**). Hematoxylin and eosin staining indicated that AZOX can decrease the cell proliferation compared to control group (**Figure 5D**). To further confirm our *in vitro* results, we detected the expression of related proteins in tumor tissues. The results showed that cleaved PARP was increased along with the cleavage of caspase-3, caspase-9 (**Figure 6A**), and caspase-8 (Supplementary Figure 1B), which was in accordance with the *in vitro* findings. Moreover, Bcl-2/Bax ratio was significantly suppressed (**Figure 6B**) along with the increased cytochrome *c* release from the mitochondria (**Figure 6A**).

## Discussion

Esophageal cancer is one of the eight most common cancers and the sixth leading cause of global cancer mortality (Li et al., 2015), with a rising incidence worldwide. According to the histological subtypes, esophageal cancer can be mainly divided into adenocarcinoma and squamous cell carcinoma. Adenocarcinoma is the dominant form in Europe while the squamous cell carcinoma is more prevalent in Asia especially in China (Cook et al., 2009; Rubenstein and Chen, 2014). Current clinical treatments for esophageal cancer include surgical resection, chemotherapy and radiation therapy (Smith et al., 1998; Gaur et al., 2014). However, patients with esophageal cancer still exhibit low 5-year survival rate (no more than 20%) and poor prognosis (Siegel et al., 2012). Seeing that the survival rate of ESCC patients remains very dismal by current chemotherapy (Ozawa et al., 2015), it is still pivotal to discover novel therapeutic compounds.

AZOX, a methoxyacrylate derived from the naturally occurring strobilurins, is commonly used as a fungicide in agriculture with low toxic side effects to mammals (Gao et al., 2014). Recent studies found that AZOX can inhibit mitochondrial respiration in metabolic cells and complex III activity in rat liver mitochondria, regulating whole-body glucose and lipid homeostasis in the development of obesity-related type 2 diabetes (Gao et al., 2014). In this study, we demonstrated the broad anti-tumor properties of AZOX in a wide variety of human cancer cell lines including esophageal, liver, and colon cancer cells. In human esophageal squamous cell carcinoma KYSE-150 cells, AZOX caused a time-dependent and dose-dependent cancer cell growth inhibition, which appears to be due to its ability to induce S-phase arrest. It is known that infinite proliferation of the tumor cells is closely associated with the cell cycle regulation (Nurse, 2000), and our results are in concordance with the previous findings that an increase of cell number in the S phase can effectively inhibit KYSE-150 cell proliferation (Li et al., 2015).

Apoptosis is a process of programmed cell death, generally characterized by distinct morphological characteristics and energy-dependent biochemical mechanisms (Elmore, 2007). The induction of apoptosis in tumor cells is known to be an important target for the therapy and prevention of cancer (Wyllie, 1985). In the present study, AZOX could induce KYSE-150 cell apoptosis in a dose- and time-dependent manner suggesting that AZOX may effectively target cancer cells by activating apoptosis pathway. The execution caspases are major indicators of the phase of apoptosis. Generally, initiator caspases (caspase-8 or caspase-9) activate the downstream effector caspase-3, subsequently cleaving PARP and causing the morphological and biochemical changes in apoptotic cells (Koh et al., 2005; Elmore, 2007). To investigate the mechanisms responsible for the selectivity induction of apoptosis by AZOX, the major components of apoptotic signaling network were analyzed. Here in our results, the active caspase-9, caspase-8, caspase-3, and PARP, were remarkably increased by AZOX in KYSE-150 cells, indicating that the apoptosis pathway is one of the anti-cancer mechanisms of AZOX.

In cancer cells, the apoptosis signaling includes intrinsic pathway and extrinsic pathway (Khan et al., 2014). The intrinsic pathway called as mitochondrial pathway is regulated by a balance of Bcl-2 superfamily proteins (Khan et al., 2014). Bcl-2 has been reported to act *in situ* on mitochondria to prevent the release of cytochrome *c* and caspase activation (Kluck et al., 1997). The imbalance of Bcl-2/Bax can induce the release of cytochrome *c* from the mitochondria, which can bind with the Apaf-1 and pro-caspase-9 to form apoptosome (Li et al., 1997). As a result, the pro-caspase-9 is transformed into cleaved caspase-9 that can further activate other caspases such as caspase-3 to initiate cell apoptosis (Green and Reed, 1998). Due to the inhibitory effect of AZOX on fungal mitochondrial pathway, we take the intrinsic apoptotic pathway into first consideration in this study. In KYSE-150 cells, the expression of Bcl-2 and Bax was slightly decreased and increased after AZOX treatment, respectively, resulting in the significant decrease of Bcl-2/Bax ratio. Meanwhile, the cytosolic level of cytochrome *c* released from mitochondria was increased by AZOX accompanied with the cleavage of caspase-9 and caspase-3. We also noticed that cleaved caspase-8 was significantly increased by AZOX after 36 h treatment. Activation of caspase-8 can both directly activate caspase-3 and affect the Bax function to initiate intrinsic pathway (Khan et al., 2014). These results indicate that the intrinsic apoptotic pathway was involved in the anticancer effect of AZOX on KYSE-150 cell line.

To further examine the anti-tumor effects and mechanisms of AZOX, the *in vivo* experiments were performed in xenograft animal model. After the treatment of AZOX, the growth of KYSE-150 esophageal xenografted tumors was significantly inhibited in the nude mice. Noticeably, AZOX administration can decrease the body weight of the nude mice compared with the control group. The reason may be due to the reduced fatty acid utilization induced by AZOX treatment as previously reported (Gao et al., 2014). Disturbance of the ratio of Bcl-2/Bax and increase of cytochrome *c* level were found in the tumor tissues after AZOX treatment, which are in accordance with the *in vitro* results. Moreover, expression of the cleaved caspase-8, caspase-9 and PARP in tumor sections were also increased by AZOX in the xenograft mice model.

## Conclusion

**FIGURE 7:**
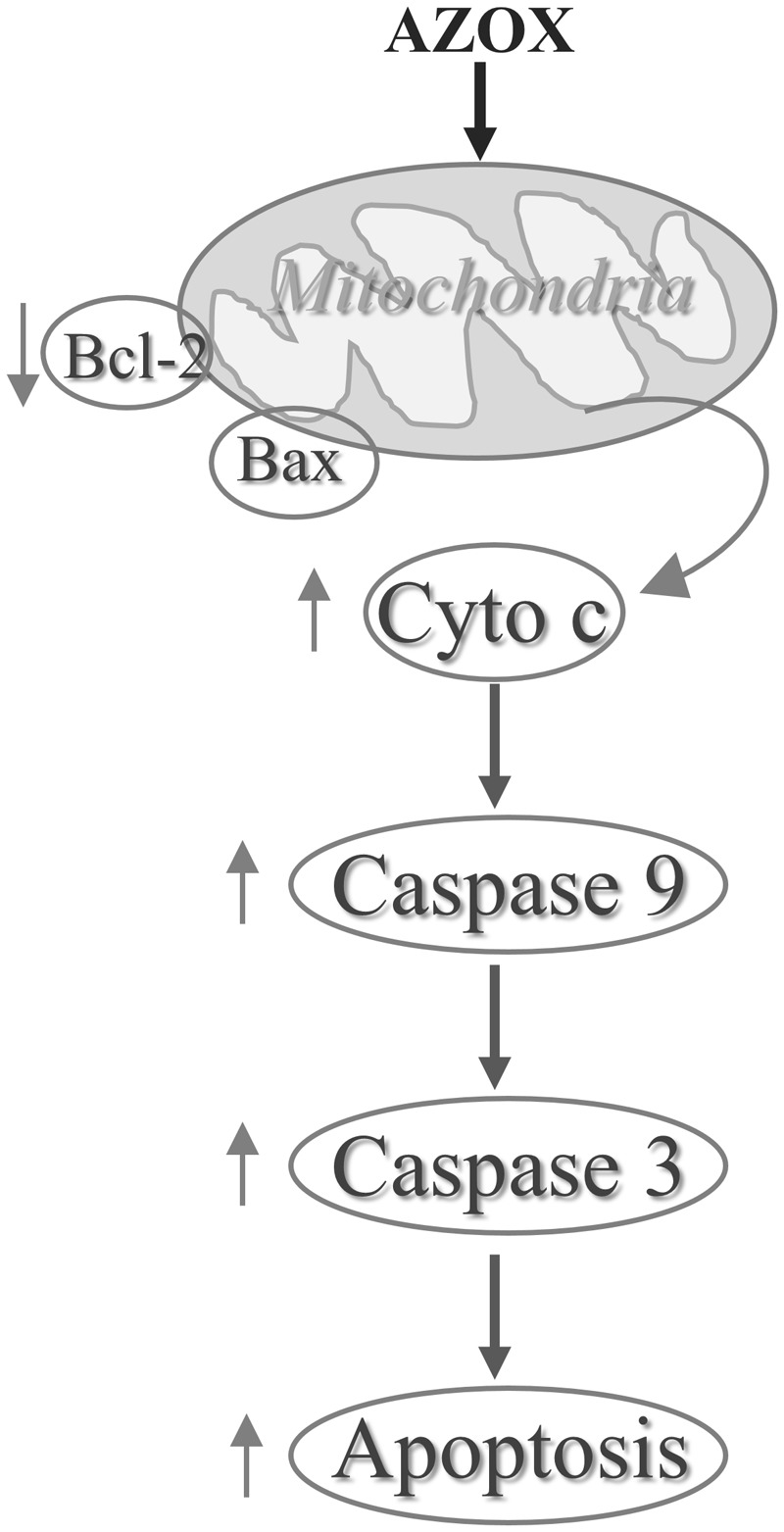
AZOX induces KYSE-150 cell apoptosis via mitochondrial pathway by suppressing Bcl-2/Bax.

In conclusion, our results show that AZOX has potent activity against the human esophageal squamous cell carcinoma KYSE-150 cell line. The effect may be mediated through Bcl-2/Bax related intrinsic mitochondrial pathway of apoptosis (**Figure 7**). AZOX or its derivatives may be developed as a novel therapy for esophageal squamous cell carcinoma.

## Author Contributions

X-kS was involved in the project design, carried out most of the experiments, and drafted the manuscript. X-bB and TH participated in the *in vitro* studies. H-xM and SF contributed to the animal experiment and data analysis. BW and LZ contributed to the western blot analysis. B-mF helped to design the experiment and contributed to critical revisions of the manuscript. C-yL, L-fH and Z-xB conceived and designed the experiment, and contributed to finalize the manuscript.

## Conflict of Interest Statement

The authors declare that the research was conducted in the absence of any commercial or financial relationships that could be construed as a potential conflict of interest.
