# Supplementary figures and images for: Azoxystrobin Induces Apoptosis of Human Esophageal Squamous Cell Carcinoma KYSE-150 Cells through Triggering of the Mitochondrial Pathway
Source: Front Pharmacol. 2017 May 17;8:277. doi: 10.3389/fphar.2017.00277 (PMC5434151; doi:10.3389/fphar.2017.00277)

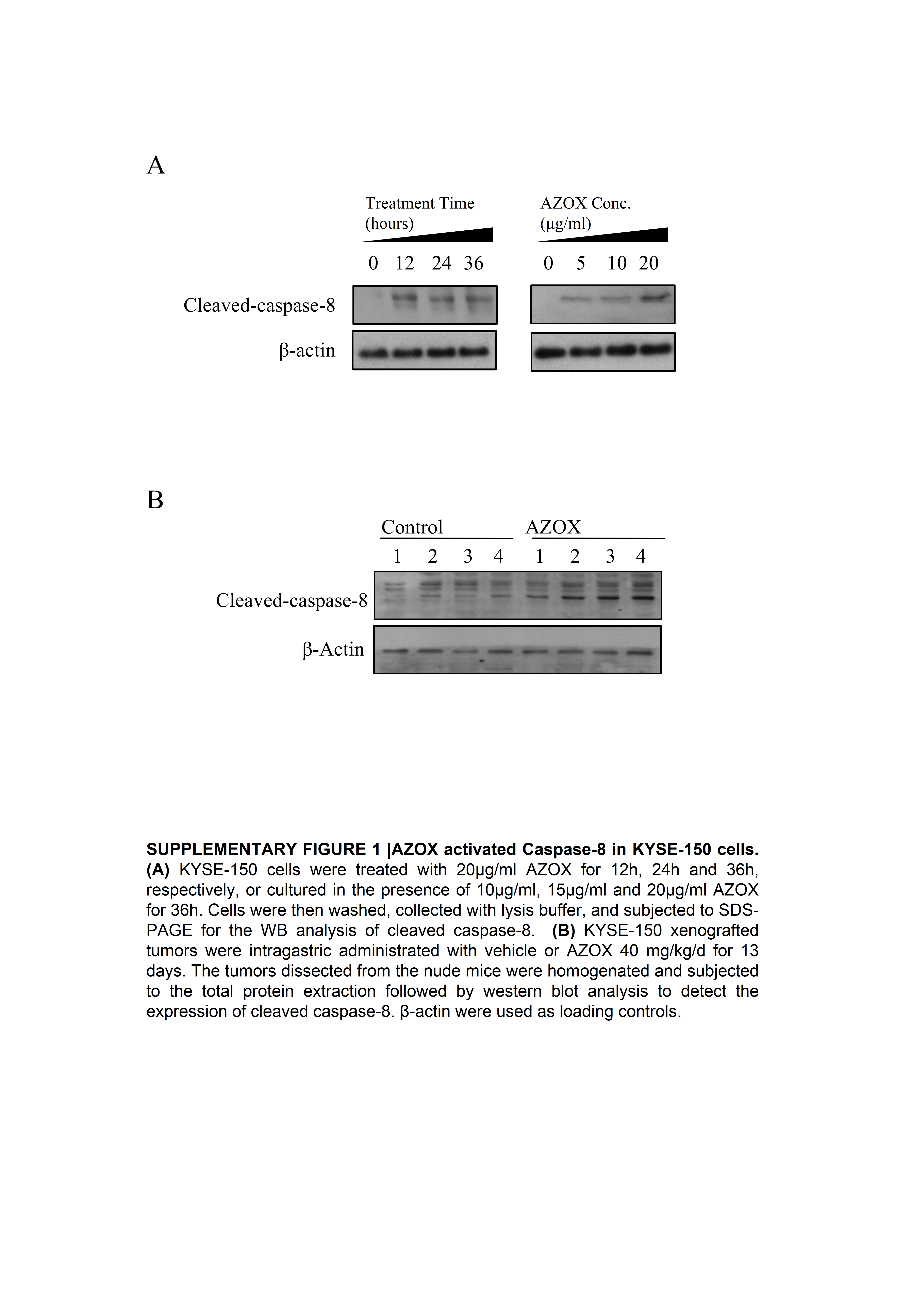

Supplement: Supplementary file 1 [file Image_1.tif]
